# P062: Commode chairs – not a “high-touch” surface but a “high-risk” surface with regard to VRE transmission

**DOI:** 10.1186/2047-2994-2-S1-P62

**Published:** 2013-06-20

**Authors:** LB van der Velden, A Voss, SM Wennekes, MJ van Mourik, MH Nabuurs-Franssen

**Affiliations:** 1 Medical Microbiology and Infectious Diseases, Canisius Wilhelmina Hospital Nijmegen, The Netherlands; 2 Medical Microbiology, University Hospital Nijmegen Medical Center, Nijmegen, The Netherlands

## Introduction

Contaminated environmental surfaces (especially high-touch surfaces), equipment, and hands of healthcare workers have been linked to the transmission of nosocomial pathogens, causing outbreaks in healthcare-settings. With regard to vancomycin-resistant enterococci (VRE), a contaminated environment seems to be of special importance. Consequently, many direct their attention to high-touch-surfaces, such as bed rails, over-bed tables, and i.v. pumps (Huslage et al). In the present study we would like to redirect the attention to a surface that is less frequently touched but was shown to be of “high-risk” during a VRE outbreak.

## Objectives

During a VRE outbreak, we determined on which environmental sources VRE can be found after terminal room cleaning after discharge of known VRE positive patients. Commode chairs and shower chairs were cultured after disinfection by instructed personnel.

## Methods

VanB, enterococcus faecium and cc17 PCRs were performed on environmental surfaces after disinfection. By this combined PCR, both vancomycin-susceptible (VSE) as well as the cc17+ VRE outbreak-strain were identified. Beds, bed bells, bedside tables and door knobs were considered high-touch surfaces, commode chairs and shower chairs were considered high-risk surfaces.

## Results

During the first evaluation of possible environmental VRE sources, both high-touch and high-risk surfaces were positive for VRE/VSE. After optimalisation of hand hygiene, increasing the compliance to contact precautions and intensifying the cleaning, high-touch surfaces were only rarely found positive for VRE/VSE. Despite these measures, VRE/VSE were still frequently found on high-risk surfaces.

## Conclusion

Our data show that the cleaning and disinfection of high-risk surfaces is more difficult than of high-touch surfaces. In controlling a VRE outbreak in which a contaminated environment is of special importance, additional measurements are needed to eliminate high-risk surfaces as an environmental VRE source.

## Disclosure of interest

None declared
